# Overdose Deaths Involving Opioids, Cocaine, and Psychostimulants — United States, 2015–2016

**DOI:** 10.15585/mmwr.mm6712a1

**Published:** 2018-03-30

**Authors:** Puja Seth, Lawrence Scholl, Rose A. Rudd, Sarah Bacon

**Affiliations:** ^1^Division of Unintentional Injury Prevention, National Center for Injury Prevention and Control, CDC; ^2^Epidemic Intelligence Service, CDC.

During 1999‒2015, 568,699 persons died from drug overdoses in the United States.* Drug overdose deaths in the United States increased 11.4% from 2014 to 2015 resulting in 52,404 deaths in 2015, including 33,091 (63.1%) that involved an opioid. The largest rate increases from 2014 to 2015 occurred among deaths involving synthetic opioids other than methadone (synthetic opioids) (72.2%) (*1*). Because of demographic and geographic variations in overdose deaths involving different drugs (*2*,*3*),^†^ CDC examined age-adjusted death rates for overdoses involving all opioids, opioid subcategories (i.e., prescription opioids, heroin, and synthetic opioids),^§^ cocaine, and psychostimulants with abuse potential (psychostimulants) by demographics, urbanization levels, and in 31 states and the District of Columbia (DC). There were 63,632 drug overdose deaths in 2016; 42,249 (66.4%) involved an opioid.^¶^ From 2015 to 2016, deaths increased across all drug categories examined. The largest overall rate increases occurred among deaths involving cocaine (52.4%) and synthetic opioids (100%), likely driven by illicitly manufactured fentanyl (IMF) (*2*,*3*). Increases were observed across demographics, urbanization levels, and states and DC. The opioid overdose epidemic in the United States continues to worsen. A multifaceted approach, with faster and more comprehensive surveillance, is needed to track emerging threats to prevent and respond to the overdose epidemic through naloxone availability, safe prescribing practices, harm-reduction services, linkage into treatment, and more collaboration between public health and public safety agencies.

Drug overdose deaths were identified in the National Vital Statistics System multiple cause-of-death mortality files,** using the *International Classification of Diseases, Tenth Revision* (ICD-10), based on ICD-10 underlying cause-of-death codes X40–44 (unintentional), X60–64 (suicide), X85 (homicide), or Y10–Y14 (undetermined intent). Among deaths with drug overdose as the underlying cause, the type of drug or drug category is indicated by the following ICD-10 multiple cause-of-death codes: opioids (T40.0, T40.1, T40.2, T40.3, T40.4, or T40.6)^††^; natural/semisynthetic opioids (T40.2); methadone (T40.3); heroin (T40.1); synthetic opioids other than methadone (T40.4); cocaine (T40.5); and psychostimulants with abuse potential (T43.6). Some deaths involved more than one type of drug; these deaths were included in the rates for each drug category. Therefore, categories are not mutually exclusive.^§§^

Age-adjusted overdose death rates^¶¶^ were examined for 2015 and 2016 for all opioids, opioid subcategories (prescription opioids [i.e., natural/semisynthetic opioids and methadone] (*4*), heroin, and synthetic opioids), cocaine, and psychostimulants in the United States and by age, sex, racial/ethnic group, urbanization level,*** and state. State-level analyses included 31 states and DC that met the following criteria: 1) ≥80% of drug overdose death certificates named at least one specific drug in 2015 and 2016; 2) change from 2015 to 2016 in the percentage of death certificates reporting at least one specific drug was <10 percentage points^†††^; and 3) ≥20 deaths occurred during 2015 and 2016 in at least two drug categories examined. These inclusion criteria were selected to ensure accurate examination of death rates and increases. Relative change in age-adjusted rates and absolute change were calculated. Significance was assessed using z-tests when the number of deaths was ≥100 (p<0.05) and nonoverlapping confidence intervals based on a gamma distribution when the number of deaths was <100.^§§§^

In the United States, 63,632 drug overdose deaths occurred in 2016; the age-adjusted rate of overdose deaths increased significantly (21.5%) from 16.3 in 2015 to 19.8 in 2016. Opioids were involved in 42,249 (66.4%) drug overdose deaths (13.3 per 100,000 population) in 2016, representing a 27.9% rate increase from 2015 (Table 1). These increases primarily were driven by deaths involving synthetic opioids, for which the rate doubled from 2015 to 2016 (Table 2). Rates of overdose deaths involving prescription opioids and heroin increased by 10.6% and 19.5%, respectively (Table 1) (Table 2), and rates of overdose deaths involving cocaine and psychostimulants increased by 52.4% and 33.3%, respectively (Table 3).

**TABLE 1:** Annual number and age-adjusted rate of drug overdose deaths* involving any opioid^†^ and prescription opioids,^§^^,^^¶^ by sex, age, race and Hispanic origin,** urbanization level,^††^ and selected states^§§^ — United States, 2015 and 2016

Decedent characteristic	Opioids						Prescription opioids					
	2015		2016		Change from 2015 to 2016^¶¶^		2015		2016		Change from 2015 to 2016^¶¶^	
	No.	Rate	No.	Rate	Absolute rate change	% Change in rate	No.	Rate	No.	Rate	Absolute rate change	% Change in rate
**All**	**33,091**	**10.4**	**42,249**	**13.3**	2.9***	27.9***	**15,281**	**4.7**	**17,087**	**5.2**	0.5***	10.6***
**Sex**												
Male	21,671	13.7	28,498	18.1	4.4***	32.1***	8,617	5.4	9,978	6.2	0.8***	14.8***
Female	11,420	7.1	13,751	8.5	1.4***	19.7***	6,664	4.0	7,109	4.3	0.3***	7.5***
**Age group (yrs)**												
0–14	83	0.1	83	0.1	0.0	0.0	61	0.1	60	0.1	0.0	0.0
15–24	3,082	7.0	4,027	9.3	2.3***	32.9***	886	2.0	1,146	2.6	0.6***	30.0***
25–34	8,568	19.4	11,552	25.9	6.5***	33.5***	2,906	6.6	3,442	7.7	1.1***	16.7***
35–44	7,484	18.4	9,747	24.1	5.7***	31.0***	3,390	8.4	3,727	9.2	0.8***	9.5***
45–54	7,595	17.6	9,074	21.2	3.6***	20.5***	4,100	9.5	4,307	10.1	0.6***	6.3***
55–64	5,089	12.4	6,321	15.2	2.8***	22.6***	3,101	7.6	3,489	8.4	0.8***	10.5***
≥65	1,188	2.5	1,441	2.9	0.4***	16.0***	835	1.7	915	1.9	0.2***	11.8***
**Sex and age group (yrs)**												
**Male**												
15–24	2,211	9.8	2,986	13.4	3.6***	36.7***	619	2.8	852	3.8	1.0***	35.7***
25–44	11,228	26.4	15,137	35.4	9.0***	34.1***	3,862	9.1	4,527	10.6	1.5***	16.5***
45–64	7,537	18.4	9,519	23.2	4.8***	26.1***	3,676	9.0	4124	10.0	1.0***	11.1***
**Female**												
15–24	871	4.1	1,041	4.9	0.8***	19.5***	267	1.2	294	1.4	0.2	16.7
25–44	4,824	11.4	6,162	14.5	3.1***	27.2***	2,434	5.8	2,642	6.2	0.4***	6.9***
45–64	5,147	12.0	5,876	13.6	1.6***	13.3***	3,525	8.2	3,672	8.5	0.3	3.7
Race and Hispanic origin**												
White, non-Hispanic	27,056	13.9	33,450	17.5	3.6***	25.9***	12,894	6.4	14,167	7.0	0.6***	9.4***
Black, non-Hispanic	2,741	6.6	4,374	10.3	3.7***	56.1***	1,060	2.6	1,392	3.3	0.7***	26.9***
Hispanic	2,507	4.6	3,440	6.1	1.5***	32.6***	961	1.8	1,133	2.1	0.3***	16.7***
AI/AN, non-Hispanic	315	12.1	369	13.9	1.8	14.9	181	7.0	173	6.5	-0.5	-7.1
A/PI, non-Hispanic	220	1.1	323	1.5	0.4***	36.4***	89	0.5	131	0.7	0.2	40
**County urbanization level^††^**												
Large central metro	9,679	9.4	12,903	12.5	3.1***	33.0***	4,276	4.1	4,930	4.7	0.6***	14.6***
Large fringe metro	8,683	11.2	11,993	15.4	4.2***	37.5***	3,444	4.2	4,209	5.2	1.0***	23.8***
Medium metro	7,618	11.8	9,264	14.3	2.5***	21.2***	3,664	5.6	3,988	6.0	0.4***	7.1***
Small metro	2,729	9.9	3,224	11.7	1.8***	18.2***	1,404	5.0	1,471	5.2	0.2	4.0
Micropolitan (nonmetro)	2,730	10.8	3,068	12.1	1.3***	12.0***	1,457	5.6	1,475	5.7	0.1	1.8
Noncore (nonmetro)	1,652	9.6	1,797	10.5	0.9***	9.4***	1,036	5.9	1,014	5.7	-0.2	-3.4
**Selected states^§§^**												
**States with very good to excellent reporting (n = 25)**												
Alaska	86	11.0	94	12.5	1.5	13.6	57	7.4	51	6.8	-0.6	-8.1
Connecticut	685	19.2	855	24.5	5.3***	27.6***	243	6.3	264	7.2	0.9	14.3
District of Columbia	98	14.5	209	30.0	15.5***	106.9***	26	3.7	66	9.3	5.6***	151.4***
Georgia	858	8.4	918	8.8	0.4	4.8	519	5.0	536	5.1	0.1	2.0
Illinois	1,381	10.7	1,947	15.3	4.6***	43.0***	351	2.7	479	3.7	1.0***	37.0***
Iowa	170	5.8	183	6.2	0.4	6.9	92	3.1	92	3.1	0.0	0.0
Maine	238	19.3	301	25.2	5.9***	30.6***	124	9.6	154	12.5	2.9***	30.2***
Maryland	1,087	17.7	1,821	29.7	12.0***	67.8***	534	8.7	812	13.1	4.4***	50.6***
Massachusetts	1,550	23.3	1,990	29.7	6.4***	27.5***	298	4.3	351	4.9	0.6	14.0
Nevada	419	13.8	408	13.3	-0.5	-3.6	298	9.8	275	8.9	-0.9	-9.2
New Hampshire	380	31.3	437	35.8	4.5	14.4	80	5.7	89	6.5	0.8	14.0
New Mexico	351	17.9	349	17.5	-0.4	-2.2	189	9.6	186	9.2	-0.4	-4.2
New York	2,166	10.8	3,009	15.1	4.3***	39.8***	895	4.4	1,100	5.4	1.0***	22.7***
North Carolina	1,171	11.9	1,506	15.4	3.5***	29.4***	635	6.4	695	6.9	0.5	7.8
Ohio	2,698	24.7	3,613	32.9	8.2***	33.2***	780	6.9	867	7.7	0.8***	11.6***
Oklahoma	427	11.2	444	11.6	0.4	3.6	328	8.6	322	8.4	-0.2	-2.3
Oregon	331	7.9	312	7.6	-0.3	-3.8	198	4.7	165	3.9	-0.8	-17.0
Rhode Island	254	23.5	279	26.7	3.2	13.6	122	10.6	114	10.5	-0.1	-0.9
South Carolina	554	11.4	628	13.1	1.7***	14.9***	361	7.3	381	7.8	0.5	6.8
Tennessee	1,038	16.0	1,186	18.1	2.1***	13.1***	693	10.5	739	11.1	0.6	5.7
Utah	448	15.9	466	16.4	0.5	3.1	385	13.7	349	12.5	-1.2	-8.8
Vermont	79	13.4	101	18.4	5.0	37.3	32	5.3	35	5.9	0.6	11.3
Virginia	820	9.9	1,130	13.5	3.6***	36.4***	322	3.8	400	4.7	0.9***	23.7***
Washington	692	9.3	709	9.4	0.1	1.1	355	4.7	388	5.0	0.3	6.4
West Virginia	629	36.0	733	43.4	7.4***	20.6***	380	21.2	340	19.7	-1.5	-7.1
**States with good reporting (n = 7)**												
Arizona	671	10.2	769	11.4	1.2***	11.8***	362	5.5	380	5.6	0.1	1.8
Colorado	495	8.7	536	9.5	0.8	9.2	288	5.1	258	4.5	-0.6	-11.8
Hawaii	62	4.1	77	5.2	1.1	26.8	42	2.8	55	3.6	0.8	28.6
Minnesota	338	6.2	396	7.4	1.2***	19.4***	177	3.2	195	3.6	0.4	12.5
Missouri	692	11.7	914	15.9	4.2***	35.9***	289	4.8	268	4.5	-0.3	-6.3
Texas	1,287	4.7	1,375	4.9	0.2	4.3	590	2.1	617	2.2	0.1	4.8
Wisconsin	622	11.2	866	15.8	4.6***	41.1***	300	5.2	382	6.7	1.5***	28.8***

**Source:** National Vital Statistics System, Mortality file.

**Abbreviations:** A/PI = Asian/Pacific Islander; AI/AN = American Indian/Alaska Native.

* Deaths are classified using the *International Classification of Diseases, Tenth Revision* (ICD-10). Drug overdose deaths are identified using underlying cause-of-death codes X40–X44, X60–X64, X85, and Y10–Y14. Rates are age-adjusted using the direct method and the 2000 U.S. standard population, except for age-specific crude rates. All rates are per 100,000 population. Cells with ≤9 deaths are not reported. Rates based on <20 deaths are not considered reliable and not reported.

^†^ Drug overdose deaths, as defined, that have opium (T40.0), heroin (T40.1), natural and semisynthetic opioids (T40.2), methadone (T40.3), synthetic opioids other than methadone (T40.4), or other and unspecified narcotics (T40.6) as a contributing cause.

^§^ Drug overdose deaths, as defined, that have natural and semisynthetic opioids (T40.2) or methadone (T40.3) as a contributing cause.

^¶^ Categories of deaths are not exclusive because deaths may involve more than one drug. Summing of categories will result in greater than the total number of deaths in a year.

** Data for Hispanic origin should be interpreted with caution; studies comparing Hispanic origin on death certificates and on census surveys have shown inconsistent reporting on Hispanic ethnicity. Potential race misclassification might lead to underestimates for certain categories, primarily AI/AN non-Hispanic and A/PI non-Hispanic decedents. https://www.cdc.gov/nchs/data/series/sr_02/sr02_172.pdf.

^††^ By 2013 urbanization classification. https://www.cdc.gov/nchs/data_access/urban_rural.htm.

^§§^ Analyses were limited to states meeting the following criteria: for states with very good to excellent reporting, ≥90% of drug overdose deaths mention at least one specific drug in 2015, with the change in drug overdose deaths mentioning at least one specific drug differing by no more than 10 percentage points from 2015 to 2016. States with good reporting had 80% to <90% of drug overdose deaths mention of at least one specific drug in 2015, with the change in the percentage of drug overdose deaths mentioning at least one specific drug differing by <10 percentage points from 2015 to 2016. States included also were required to have stable rate estimates, based on ≥20 deaths, in at least two drug categories (i.e., opioids, prescription opioids, synthetic opioids other than methadone, heroin, cocaine, and psychostimulants with abuse potential). South Dakota was the only state with good or excellent reporting in both years, but with an improvement >10 percentage points in drug specificity.

^¶¶^ Absolute rate change is the difference between 2015 and 2016 rates. Percent change is the absolute rate change divided by the 2015 rate, multiplied by 100. Nonoverlapping confidence intervals based on the gamma method were used if the number of deaths was <100 in 2015 or 2016, and z-tests were used if the number of deaths was ≥100 in both 2015 and 2016. Note that the method of comparing confidence intervals is a conservative method for statistical significance; caution should be observed when interpreting a nonsignificant difference when the lower and upper limits being compared overlap only slightly. Confidence intervals of 2015 and 2016 rates of prescription opioid deaths for Asian/Pacific Islanders overlapped only slightly: (0.37, 0.57), (0.56, 0.80).

*** Statistically significant at 0.05 level.

**TABLE 2:** Annual number and age-adjusted rate of drug overdose deaths* involving heroin^†^ and synthetic opioids other than methadone,^§^^,^^¶^ by sex, age, race and Hispanic origin,** urbanization level,^††^ and selected states^§§^ — United States, 2015 and 2016

Decedent characteristic	Heroin						Synthetic opioids other than methadone					
	2015		2016		Change from 2015 to 2016^¶¶^		2015		2016		Change from 2015 to 2016^¶¶^	
	No.	Rate	No.	Rate	Absolute rate change	% Change in rate	No.	Rate	No.	Rate	Absolute rate change	% Change in rate
**All**	**12,989**	**4.1**	**15,469**	**4.9**	0.8***	19.5***	**9,580**	**3.1**	**19,413**	**6.2**	3.1***	100.0***
**Sex**												
Male	9,881	6.3	11,752	7.5	1.2***	19.0***	6,560	4.2	13,835	8.9	4.7***	111.9***
Female	3,108	2.0	3,717	2.4	0.4***	20.0***	3,020	1.9	5,578	3.5	1.6***	84.2***
**Age group (yrs)**												
0–14	—^†††^	—^†††^	—^†††^	—^†††^	—^†††^	—^†††^	14	—^†††^	18	—^†††^	—^†††^	—^†††^
15–24	1,649	3.8	1,728	4.0	0.2	5.3	999	2.3	1,958	4.5	2.2***	95.7***
25–34	4,292	9.7	5,051	11.3	1.6***	16.5***	2,896	6.6	6,094	13.6	7.0***	106.1***
35–44	3,012	7.4	3,625	9.0	1.6***	21.6***	2,289	5.6	4,825	11.9	6.3***	112.5***
45–54	2,439	5.6	3,009	7.0	1.4***	25.0***	1,982	4.6	3,872	9.1	4.5***	97.8***
55–64	1,407	3.4	1,777	4.3	0.9***	26.5***	1,167	2.9	2,238	5.4	2.5***	86.2***
≥65	184	0.4	275	0.6	0.2***	50.0***	232	0.5	405	0.8	0.3***	60.0***
**Sex and age group (yrs)**												
**Male**												
15–24	1,172	5.2	1,275	5.7	0.5***	9.6***	718	3.2	1,434	6.4	3.2***	100.0***
25–44	5,602	13.2	6,643	15.5	2.3***	17.4***	3,764	8.9	8,029	18.8	9.9***	111.2***
45–64	2,953	7.2	3,599	8.8	1.6***	22.2***	1,948	4.8	4,116	10.0	5.2***	108.3***
**Female**												
15–24	477	2.2	453	2.1	-0.1	-4.5	281	1.3	524	2.5	1.2***	92.3***
25–44	1,702	4.0	2,033	4.8	0.8***	20.0***	1,421	3.4	2,890	6.8	3.4***	100.0***
45–64	893	2.1	1,187	2.8	0.7***	33.3***	1,201	2.8	1,994	4.6	1.8***	64.3***
Race and Hispanic origin**												
White, non-Hispanic	10,050	5.4	11,631	6.3	0.9***	16.7***	7,995	4.2	15,143	8.2	4.0***	95.2***
Black, non-Hispanic	1,310	3.1	1,899	4.5	1.4***	45.2***	883	2.1	2,391	5.6	3.5***	166.7***
Hispanic	1,299	2.3	1,555	2.8	0.5***	21.7***	524	0.9	1,505	2.7	1.8***	200.0***
AI/AN, non-Hispanic	117	4.4	131	5.0	0.6	13.6	51	2.0	113	4.1	2.1***	105.0***
A/PI, non-Hispanic	98	0.5	102	0.5	0.0	0.0	51	0.2	134	0.6	0.4***	200.0***
**County urbanization level^††^**												
Large central metro	4,496	4.4	5,507	5.3	0.9***	20.5***	2,509	2.4	6,009	5.8	3.4***	141.7***
Large fringe metro	3,778	5.0	4,623	6.1	1.1***	22.0***	2,947	3.9	6,264	8.2	4.3***	110.3***
Medium metro	2,736	4.3	3,077	4.9	0.6***	14.0***	2,255	3.5	3,978	6.3	2.8***	80.0***
Small metro	868	3.2	990	3.7	0.5***	15.6***	686	2.5	1,270	4.7	2.2***	88.0***
Micropolitan (nonmetro)	778	3.2	860	3.6	0.4***	12.5***	753	3.0	1,228	5.0	2.0***	66.7***
Noncore (nonmetro)	333	2.1	412	2.6	0.5***	23.8***	430	2.6	664	4.1	1.5***	57.7***
**Selected states^§§^**												
**States with very good to excellent reporting (n = 25)**												
Alaska	37	4.7	49	6.5	1.8	38.3	14	—^†††^	—^†††^	—^†††^	—^†††^	—^†††^
Connecticut	390	11.3	450	13.1	1.8***	15.9***	211	6.1	500	14.8	8.7***	142.6***
District of Columbia	67	9.9	122	17.3	7.4***	74.7***	26	3.9	129	19.2	15.3***	392.3***
Georgia	222	2.2	226	2.2	0.0	0.0	284	2.8	277	2.7	-0.1	-3.6
Illinois	844	6.7	1,040	8.2	1.5***	22.4***	278	2.2	907	7.2	5.0***	227.3***
Iowa	45	1.6	47	1.7	0.1	6.2	44	1.5	58	2.0	0.5	33.3
Maine	52	4.5	55	4.7	0.2	4.4	116	9.9	199	17.3	7.4***	74.7***
Maryland	405	6.6	650	10.7	4.1***	62.1***	357	5.8	1,091	17.8	12.0***	206.9***
Massachusetts	634	9.6	630	9.5	-0.1	-1.0	949	14.4	1,550	23.5	9.1***	63.2***
Nevada	82	2.7	86	2.9	0.2	7.4	32	1.1	53	1.7	0.6	54.5
New Hampshire	78	6.5	34	2.8	-3.7***	-56.9***	285	24.1	363	30.3	6.2***	25.7***
New Mexico	156	8.1	161	8.2	0.1	1.2	42	2.1	78	4.0	1.9***	90.5***
New York	1,058	5.4	1,307	6.5	1.1***	20.4***	668	3.3	1,641	8.3	5.0***	151.5***
North Carolina	393	4.1	544	5.7	1.6***	39.0***	300	3.1	601	6.2	3.1***	100.0***
Ohio	1,444	13.3	1,478	13.5	0.2	1.5	1,234	11.4	2,296	21.1	9.7***	85.1***
Oklahoma	36	1.0	53	1.4	0.4	40.0	93	2.4	98	2.5	0.1	4.2
Oregon	102	2.5	114	2.9	0.4	16.0	34	0.9	43	1.1	0.2	22.2
Rhode Island	45	4.3	25	2.5	-1.8	-41.9	137	13.2	182	17.8	4.6***	34.8***
South Carolina	100	2.2	115	2.5	0.3	13.6	161	3.3	237	5.0	1.7***	51.5***
Tennessee	205	3.3	260	4.1	0.8***	24.2***	251	4.0	395	6.2	2.2***	55.0***
Utah	127	4.3	166	5.6	1.3***	30.2***	62	2.3	72	2.5	0.2	8.7
Vermont	33	5.8	45	8.7	2.9	50.0	33	5.6	53	10.1	4.5	80.4
Virginia	353	4.3	450	5.5	1.2***	27.9***	270	3.3	648	7.9	4.6***	139.4***
Washington	303	4.2	283	3.9	-0.3	-7.1	65	0.9	93	1.3	0.4	44.4
West Virginia	194	11.8	235	14.9	3.1***	26.3***	217	12.7	435	26.3	13.6***	107.1***
**States with good reporting (n = 7)**												
Arizona	247	3.8	299	4.5	0.7***	18.4***	72	1.1	123	1.8	0.7***	63.6***
Colorado	159	2.8	234	4.2	1.4***	50.0***	64	1.2	72	1.3	0.1	8.3
Hawaii	15	—^†††^	20	1.4	—^†††^	—^†††^	13	—^†††^	—^†††^	—^†††^	—^†††^	—^†††^
Minnesota	115	2.2	149	2.8	0.6	27.3	55	1.0	99	1.9	0.9***	90.0***
Missouri	303	5.3	380	6.7	1.4***	26.4***	183	3.1	441	7.8	4.7***	151.6***
Texas	523	1.9	530	1.9	0.0	0.0	186	0.7	250	0.9	0.2***	28.6***
Wisconsin	287	5.3	389	7.3	2.0***	37.7***	112	2.1	288	5.3	3.2***	152.4***

**Source:** National Vital Statistics System, Mortality file.

**Abbreviations:** A/PI = Asian/Pacific Islander; AI/AN = American Indian/Alaska Native.

* Deaths are classified using the *International Classification of Diseases, Tenth Revision* (ICD-10). Drug overdose deaths are identified using underlying cause-of-death codes X40–X44, X60–X64, X85, and Y10–Y14. Rates are age-adjusted using the direct method and the 2000 U.S. standard population, except for age-specific crude rates. All rates are per 100,000 population.

^†^ Drug overdose deaths, as defined, that have heroin (T40.1) as a contributing cause.

^§^ Drug overdose deaths, as defined, that have synthetic opioids other than methadone (T40.4) as a contributing cause.

^¶^ Categories of deaths are not exclusive because deaths may involve more than one drug. Summing of categories will result in greater than the total number of deaths in a year.

** Data for Hispanic origin should be interpreted with caution; studies comparing Hispanic origin on death certificates and on census surveys have shown inconsistent reporting on Hispanic ethnicity. Potential race misclassification might lead to underestimates for certain categories, primarily AI/AN non-Hispanic and A/PI non-Hispanic decedents. https://www.cdc.gov/nchs/data/series/sr_02/sr02_172.pdf.

^††^ By 2013 urbanization classification. https://www.cdc.gov/nchs/data_access/urban_rural.htm.

^§§^ Analyses were limited to states meeting the following criteria: For states with very good to excellent reporting, ≥90% of drug overdose deaths mention at least one specific drug in 2015, with the change in drug overdose deaths mentioning at least one specific drug differing by <10 percentage points from 2015 to 2016. States with good reporting had 80% to <90% of drug overdose deaths mention of at least one specific drug in 2015, with the change in the percentage of drug overdose deaths mentioning at least one specific drug differing by <10 percentage points from 2015 to 2016. States included also were required to have stable rate estimates, based on ≥20 deaths, in at least two drug categories (i.e., opioids, prescription opioids, synthetic opioids other than methadone, heroin, cocaine, and psychostimulants with abuse potential). South Dakota was the only state with good or excellent reporting in both years, but with an improvement >10 percentage points in drug specificity.

^¶¶^ Absolute rate change is the difference between 2015 and 2016 rates. Percent change is the absolute rate change divided by the 2015 rate, multiplied by 100. Nonoverlapping confidence intervals based on the gamma method were used if the number of deaths was <100 in 2015 or 2016, and z-tests were used if the number of deaths was ≥100 in both 2015 and 2016.

*** Statistically significant at 0.05 level.

^†††^ Cells with ≤9 deaths are not reported. Rates based on <20 deaths are not considered reliable and not reported.

**TABLE 3:** Annual number and age-adjusted rate of drug overdose deaths* involving cocaine^†^ and psychostimulants with abuse potential,^§^^,^^¶^ by sex, age, race and Hispanic origin,** urbanization level,^††^ and selected states^§§^ — United States, 2015 and 2016

Decedent characteristic	Cocaine						Psychostimulants with abuse potential					
	2015		2016		Change from 2015 to 2016^¶¶^		2015		2016		Change from 2015 to 2016^¶¶^	
	No.	Rate	No.	Rate	Absolute rate change	% Change in rate	No.	Rate	No.	Rate	Absolute rate change	% Change in rate
**All**	**6,784**	**2.1**	**10,375**	**3.2**	1.1***	52.4***	**5,716**	**1.8**	**7,542**	**2.4**	0.6***	33.3***
**Sex**												
Male	4,885	3.1	7,493	4.7	1.6***	51.6***	3,971	2.5	5,348	3.4	0.9***	36.0***
Female	1,899	1.2	2,882	1.8	0.6***	50.0***	1,745	1.1	2,194	1.4	0.3***	27.3***
**Age group (yrs)**												
0–14	—^†††^	—^†††^	—^†††^	—^†††^	—^†††^	—^†††^	11	—^†††^	11	—^†††^	—^†††^	—^†††^
15–24	442	1.0	757	1.7	0.7***	70.0***	416	0.9	571	1.3	0.4***	44.4***
25–34	1,571	3.6	2,525	5.7	2.1***	58.3***	1,307	3.0	1,762	3.9	0.9***	30.0***
35–44	1,549	3.8	2,431	6.0	2.2***	57.9***	1,357	3.3	1,831	4.5	1.2***	36.4***
45–54	1,861	4.3	2,629	6.1	1.8***	41.9***	1,513	3.5	1,914	4.5	1.0***	28.6***
55–64	1,166	2.9	1,721	4.2	1.3***	44.8***	946	2.3	1,244	3.0	0.7***	30.4***
≥65	194	0.4	303	0.6	0.2***	50.0***	164	0.3	206	0.4	0.1***	33.3***
**Sex and age group (yrs)**												
**Male**												
15–24	303	1.3	553	2.5	1.2***	92.3***	259	1.2	388	1.7	0.5***	41.7***
25–44	2,238	5.3	3,569	8.3	3.0***	56.6***	1,853	4.4	2,536	5.9	1.5***	34.1***
45–64	2,181	5.3	3,108	7.6	2.3***	43.4***	1,714	4.2	2,251	5.5	1.3***	31.0***
**Female**												
15–24	139	0.7	204	1.0	0.3***	42.9***	157	0.7	183	0.9	0.2***	28.6***
25–44	882	2.1	1,387	3.3	1.2***	57.1***	811	1.9	1,057	2.5	0.6***	31.6***
45–64	846	2.0	1,242	2.9	0.9***	45.0***	745	1.7	907	2.1	0.4***	23.5***
Race and Hispanic origin**												
White, non-Hispanic	4,225	2.2	6,443	3.4	1.2***	54.5***	4,324	2.2	5,777	3.0	0.8***	36.4***
Black, non-Hispanic	1,690	4.0	2,599	6.1	2.1***	52.5***	316	0.8	477	1.2	0.4***	50.0***
Hispanic	697	1.3	1,097	2.0	0.7***	53.8***	725	1.4	846	1.5	0.1	7.1
AI/AN, non-Hispanic	43	1.6	56	2.1	0.5	31.3	142	5.4	181	6.9	1.5***	27.8***
A/PI, non-Hispanic	61	0.3	85	0.4	0.1	33.3	149	0.7	171	0.8	0.1	14.3
**County urbanization level^††^**												
Large central metro	2,786	2.7	4,301	4.2	1.5***	55.6***	2,003	2.0	2,561	2.5	0.5***	25.0***
Large fringe metro	1,617	2.1	2,734	3.5	1.4***	66.7***	909	1.2	1,235	1.6	0.4***	33.3***
Medium metro	1,462	2.3	2,082	3.2	0.9***	39.1***	1,378	2.1	1,821	2.8	0.7***	33.3***
Small metro	419	1.5	569	2.1	0.6***	40.0***	533	2.0	698	2.6	0.6***	30.0***
Micropolitan (nonmetro)	360	1.4	474	1.9	0.5***	35.7***	517	2.0	745	3.0	1.0***	50.0***
Noncore (nonmetro)	140	0.9	215	1.3	0.4***	44.4***	376	2.3	482	2.9	0.6***	26.1***
**Selected states^§§^**												
**States with very good to excellent reporting (n = 25)**												
Alaska	—^†††^	—^†††^	15	—^†††^	—^†††^	—^†††^	27	3.5	49	6.3	2.8	80.0
Connecticut	166	4.7	237	6.9	2.2***	46.8***	22	0.6	25	0.7	0.1	16.7
District of Columbia	33	4.9	89	13.5	8.6***	175.5***	—^†††^	—^†††^	—^†††^	—^†††^	—^†††^	—^†††^
Georgia	159	1.5	209	2.0	0.5***	33.3***	220	2.2	243	2.4	0.2	9.1
Illinois	332	2.5	507	4.0	1.5***	60.0***	60	0.5	112	0.9	0.4***	80.0***
Iowa	17	—^†††^	15	—^†††^	—^†††^	—^†††^	63	2.2	80	2.7	0.5	22.7
Maine	32	2.8	61	5.0	2.2***	78.6***	21	1.7	28	2.3	0.6	35.3
Maryland	143	2.3	314	5.0	2.7***	117.4***	26	0.4	43	0.8	0.4	100.0
Massachusetts	402	6.1	567	8.5	2.4***	39.3***	43	0.6	45	0.7	0.1	16.7
Nevada	40	1.3	37	1.2	-0.1	-7.7	172	5.7	228	7.5	1.8***	31.6***
New Hampshire	47	4.1	61	5.0	0.9	22.0	—^†††^	—^†††^	13	—^†††^	—^†††^	—^†††^
New Mexico	51	2.6	58	3.0	0.4	15.4	119	6.1	135	7.1	1.0	16.4
New York	634	3.1	991	4.9	1.8***	58.1***	80	0.4	150	0.8	0.4***	100.0***
North Carolina	314	3.2	500	5.1	1.9***	59.4***	67	0.7	115	1.2	0.5***	71.4***
Ohio	698	6.3	1,124	10.1	3.8***	60.3***	105	1.0	243	2.3	1.3***	130.0***
Oklahoma	29	0.7	31	0.8	0.1	14.3	199	5.3	263	7.1	1.8***	34.0***
Oregon	22	0.6	26	0.7	0.1	16.7	124	3.1	150	3.6	0.5	16.1
Rhode Island	87	8.3	112	10.7	2.4	28.9	11	—^†††^	10	—^†††^	—^†††^	—^†††^
South Carolina	116	2.4	143	3.0	0.6	25.0	87	1.9	125	2.7	0.8	42.1
Tennessee	202	3.0	249	3.8	0.8***	26.7***	113	1.8	186	2.9	1.1***	61.1***
Utah	44	1.5	48	1.7	0.2	13.3	147	5.2	143	5.1	-0.1	-1.9
Vermont	14	—^†††^	21	4.0	—^†††^	—^†††^	—^†††^	—^†††^	—^†††^	—^†††^	—^†††^	—^†††^
Virginia	168	2.0	254	3.0	1.0***	50.0***	55	0.7	76	0.9	0.2	28.6
Washington	85	1.1	90	1.2	0.1	9.1	304	4.2	326	4.4	0.2	4.8
West Virginia	94	5.6	143	8.5	2.9***	51.8***	65	3.9	117	7.0	3.1***	79.5***
**States with good reporting (n = 7)**												
Arizona	62	0.9	82	1.2	0.3	33.3	333	5.1	454	6.7	1.6***	31.4***
Colorado	60	1.0	106	1.9	0.9***	90.0***	140	2.6	200	3.6	1.0***	38.5***
Hawaii	—^†††^	—^†††^	—^†††^	—^†††^	—^†††^	—^†††^	87	5.9	102	6.8	0.9	15.3
Minnesota	42	0.7	43	0.8	0.1	14.3	82	1.5	140	2.6	1.1***	73.3***
Missouri	77	1.3	103	1.8	0.5	38.5	133	2.4	185	3.3	0.9***	37.5***
Texas	470	1.7	584	2.1	0.4***	23.5***	454	1.7	577	2.1	0.4***	23.5***
Wisconsin	115	2.0	147	2.6	0.6***	30.0***	38	0.7	76	1.4	0.7***	100.0***

**Source:** National Vital Statistics System, Mortality file.

**Abbreviations:** A/PI = Asian/Pacific Islander; AI/AN = American Indian/Alaska Native.

* Deaths are classified using the *International Classification of Diseases, Tenth Revision* (ICD-10). Drug overdose deaths are identified using underlying cause-of-death codes X40–X44, X60–X64, X85, and Y10–Y14. Rates are age-adjusted using the direct method and the 2000 U.S. standard population, except for age-specific crude rates. All rates are per 100,000 population.

^†^ Drug overdose deaths, as defined, that have cocaine (T40.5) as a contributing cause.

^§^ Drug overdose deaths, as defined, that have psychostimulants with abuse potential (T43.6) as a contributing cause.

^¶^ Categories of deaths are not exclusive because deaths may involve more than one drug. Summing of categories will result in greater than the total number of deaths in a year.

** Data for Hispanic origin should be interpreted with caution; studies comparing Hispanic origin on death certificates and on census surveys have shown inconsistent reporting on Hispanic ethnicity. Potential race misclassification might lead to underestimates for certain categories, primarily AI/AN non-Hispanic and A/PI non-Hispanic decedents. https://www.cdc.gov/nchs/data/series/sr_02/sr02_172.pdf.

^††^ By 2013 urbanization classification. https://www.cdc.gov/nchs/data_access/urban_rural.htm.

^§§^ Analyses were limited to states meeting the following criteria: For states with very good to excellent reporting, ≥90% of drug overdose deaths mention at least one specific drug in 2015, with the change in drug overdose deaths mentioning at least one specific drug differing by <10 percentage points from 2015 to 2016. States with good reporting had 80% to <90% of drug overdose deaths mention of at least one specific drug in 2015, with the change in the percentage of drug overdose deaths mentioning at least one specific drug differing by <10 percentage points from 2015 to 2016. States included also were required to have stable rate estimates, based on ≥20 deaths, in at least two drug categories (i.e., opioids, prescription opioids, synthetic opioids other than methadone, heroin, cocaine, and psychostimulants with abuse potential). South Dakota was the only state with good or excellent reporting in both years, but with an improvement >10 percentage points in drug specificity.

^¶¶^ Absolute rate change is the difference between 2015 and 2016 rates. Percent change is the absolute rate change divided by the 2015 rate, multiplied by 100. Nonoverlapping confidence intervals based on the gamma method were used if the number of deaths was <100 in 2015 or 2016, and z-tests were used if the number of deaths was ≥100 in both 2015 and 2016.

*** Statistically significant at 0.05 level.

^†††^ Cells with ≤9 deaths are not reported. Rates based on <20 deaths are not considered reliable and not reported.

From 2015 to 2016, opioid-involved deaths increased in males and females and among persons aged ≥15 years, whites, blacks, Hispanics, and Asian/Pacific Islanders. The largest relative rate change occurred among blacks (56.1%) (Table 1). The largest absolute rate increases of opioid-involved deaths and deaths involving synthetic opioids occurred among males aged 25–44 years and persons aged 25–34 years. However, deaths involving synthetic opioids increased in every subgroup examined (Table 2). Rates involving prescription opioids, heroin, cocaine, and psychostimulants increased for both sexes, whites, blacks, and most age groups (Table 1) (Table 2) (Table 3). Counties in large central and fringe metro areas experienced the largest absolute increases in deaths involving prescription and synthetic opioids, heroin, and cocaine; micropolitan areas experienced the largest increase in rates involving psychostimulants (Table 1) (Table 2) (Table 3).

Opioid death rates differed across the 31 states and DC, with synthetic opioids driving increases in many states.^¶¶¶^ Although several states experienced increases across drug categories, in many, the changes from 2015 to 2016 were not significant. Rates of deaths involving synthetic opioids ranged from 0.9 to 30.3 per 100,000, with the largest rates and increases concentrated in eastern states. New Hampshire (30.3 per 100,000), West Virginia (26.3), and Massachusetts (23.5) had the highest synthetic opioid death rates. Twenty states and DC experienced increases in overdose death rates involving synthetic opioids, with 10 experiencing increases by ≥100%; the largest such increase (392.3%) occurred in DC, followed by Illinois (227.3%) and Maryland (206.9%) (Table 2). Many states with large increases in synthetic opioid death rates also had large increases in rates involving other drug categories (e.g., Maryland, Virginia, and DC), including any opioid, prescription opioids (Table 1), heroin (Table 2), and cocaine (Table 3).

Thirteen states and DC experienced significant increases in heroin-involved death rates, whereas a significant decrease (56.9%) occurred in New Hampshire (Table 2). In 2016, the highest rates were in DC (17.3 per 100,000), West Virginia (14.9), and Ohio (13.5). The rates of prescription opioid–involved overdose deaths significantly increased in seven states and DC, with the highest rates in West Virginia (19.7), Maryland (13.1), Maine (12.5), and Utah (12.5) (Table 1). The highest cocaine-involved overdose death rates occurred in DC (13.5), Rhode Island (10.7), and Ohio (10.1), with 15 states and DC experiencing a significant increase from 2015 (Table 3). Significant increases in overdose death rates from heroin, prescription opioids, and cocaine occurred primarily in states in the eastern part of the country. Fourteen states experienced significant increases in psychostimulant-involved overdose death rates. The highest rates were in midwestern and western states: Nevada (7.5), New Mexico (7.1), and Oklahoma (7.1) (Table 3).

## Discussion

Drug overdoses resulted in 632,331 deaths from 1999 to 2016 in the United States, with 351,630 being opioid overdose deaths.**** The epidemic has continued to worsen, with deaths increasing from 2015 to 2016 across all drug categories examined. Opioid-involved overdoses accounted for two thirds of drug overdose deaths, with increases across age and racial/ethnic groups, urbanization levels, and in numerous states. The findings highlight wide state and regional variations. Some states (e.g., New Hampshire, Ohio, and West Virginia,) experienced the highest overdose death rates across multiple drug categories, and others (primarily in the Midwest and West) recorded the highest rates of psychostimulant-involved overdose deaths. In New Hampshire, although heroin-involved death rates declined from 2015 to 2016, deaths involving synthetic opioids increased, as they did in most states. In addition, in some states (e.g., Maryland, Rhode Island, and West Virginia), 2016 rates of prescription opioid–involved deaths were higher than were those involving heroin. These data highlight the persistent and multifaceted nature of overdoses.

The first wave of opioid overdose deaths began in the 1990s and included prescription opioid deaths.^††††^ A second wave, which began in 2010, was characterized by heroin deaths (*5*). A third wave started in 2013, with deaths involving highly potent synthetic opioids, particularly IMF and fentanyl analogs (*2*,*3*,*6*).^§§§§^ Synthetic opioid-involved deaths in 2016 accounted for 30.5% of all drug overdose deaths and 45.9% of all opioid-involved deaths, with a 100% increase in the rate of these deaths compared with 2015. Synthetic opioids propelled increases with 19,413 deaths (more than any drug examined), and previous findings underscore the contribution of IMF. In addition, IMF is now being mixed into counterfeit opioid and benzodiazepine pills, heroin, and cocaine, likely contributing to increases in overdose death rates involving other substances (*3*,*7*,*8*).

The findings in this report are subject to at least five limitations. First, at autopsy, substances tested for, and circumstances under which tests are performed to determine which drugs are present, vary by time and jurisdiction, and improvements in toxicologic testing might account for some reported increases. Second, 17% (2015) and 15% (2016) of drug overdose death certificates did not include the specific types of drugs involved, and the percentage of drug overdose death certificates with at least one drug specified varied widely by state, ranging from 52.5% to 99.3% in 2016. This variation limits rate comparisons between states. Third, because heroin and morphine are metabolized similarly (*9*), some heroin deaths might have been misclassified as morphine deaths, resulting in underreporting of heroin deaths. Fourth, potential race misclassification might lead to underestimates for certain categories, primarily for American Indian/Alaska Natives and Asian/Pacific Islanders.^¶¶¶¶^ Finally, state-specific analyses are restricted to 31 states and DC, limiting generalizability.

The ongoing and worsening drug overdose epidemic requires immediate attention and action. Faster access to data collected is needed to understand emerging threats in local communities and to tailor response activities. CDC’s Enhanced State Opioid Overdose Surveillance program funds 32 states and DC for more timely and comprehensive nonfatal and fatal overdose data, including funding for improved comprehensive toxicologic testing to identify emerging drug threats in opioid-involved fatal overdoses.***** Syndromic surveillance data allow communities to identify overdoses quickly (*10*). The State Unintentional Drug Overdose Reporting System provides improved collection of toxicology data to identify specific drugs involved (*6*), information gathered from death scene investigations, and risk factors associated with fatal overdoses. Given the continuing threat from prescription opioids and the evolving threat from illicit opioids and other substances, a multifaceted prevention approach is required. Efforts to ensure safe prescribing practices^†††††^ are enhanced by access to nonopioid and nonpharmacologic treatments for pain. Other important efforts include increasing naloxone availability, expanding access to medication-assisted treatment, and maximizing the ability of health systems to link persons to treatment and harm reduction services (*10*). CDC supports many of these efforts through the Prevention for States and Data-Driven Prevention Initiatives,^§§§§§^ which together support opioid overdose prevention efforts in 42 states and DC. Collaboration with law enforcement, first responders, and harm reduction partners is also important to understanding local variations in drug supply and lethality and to implementing a multisectoral prevention approach.

**Summary**

**What is already known about this topic?**

From 1999 to 2015, the drug overdose epidemic resulted in approximately 568,699 deaths. In 2015, 52,404 drug overdose deaths occurred; 63.1% (33,091) involved an opioid. From 2014 to 2015, the age-adjusted opioid-involved death rate increased by 15.6%; the rapid increase in deaths was driven in large part by synthetic opioids other than methadone (e.g., fentanyl).

**What is added by this report?**

In 2016, there were 63,632 drug overdose deaths in the United States. Opioids accounted for 66.4% (42,249) of deaths, with increases across age groups, racial/ethnic groups, urbanization levels, and multiple states. Age-adjusted death rates for overdoses involving synthetic opioids other than methadone doubled from 2015 to 2016, and death rates from prescription opioids, heroin, cocaine, and psychostimulants also increased.

**What are the implications for public health practice?**

There is an urgent need to implement a multifaceted, collaborative public health and public safety approach. Building on existing resources, more rapidly available and comprehensive surveillance data are needed to track emerging drug threats to guide public action to prevent and respond to the epidemic through increased naloxone availability, harm reduction services, linkage into treatment (including medication-assisted treatment), safe prescribing practices, and supporting law enforcement strategies to reduce the illicit drug supply.

## Footnotes

*
https://wonder.cdc.gov .

†
https://www.cdc.gov/drugoverdose/pdf/pubs/2017-cdc-drug-surveillance-report.pdf .

§ Natural opioids include morphine and codeine, and semisynthetic opioids include drugs such as oxycodone, hydrocodone, hydromorphone, and oxymorphone. Methadone is a synthetic opioid. Synthetic opioids, other than methadone, include drugs such as tramadol and fentanyl. Heroin is an illicit opioid synthesized from morphine that can be a white or brown powder, or a black sticky substance.

¶
https://www.cdc.gov/nchs/products/databriefs/db294.htm .

** https://www.cdc.gov/nchs/nvss/mortality_public_use_data.htm.

†† T40.0 (opium) and T40.6 (other and unspecified narcotics).

§§ For example, a death involving both a synthetic opioid other than methadone and heroin would be included in both the synthetic other than methadone and heroin death rates.

¶¶ Age-adjusted death rates were calculated by applying age-specific death rates to the 2000 U.S. Census standard population age distribution. https://www.cdc.gov/nchs/data/nvsr/nvsr61/nvsr61_04.pdf.

*** Categories of 2013 NCHS Urban-Rural Classification Scheme for Counties (https://www.cdc.gov/nchs/data_access/urban_rural.htm): *Large central metro*: Counties in metropolitan statistical areas (MSAs) of ≥1 million population that 1) contain the entire population of largest principal city of the MSA, or 2) have their entire population contained in the largest principal city of the MSA, or 3) contain at least 250,000 inhabitants of any principal city of the MSA; *Large fringe metro*: Counties in MSAs of ≥1 million population that did not qualify as large central metro counties; *Medium metro*: Counties in MSAs of populations of 250,000–999,999; *Small metro*: Counties in MSAs of populations <250,000; *Micropolitan* (nonmetropolitan counties): counties in micropolitan statistical areas; *Noncore* (nonmetropolitan counties): nonmetropolitan counties that did not qualify as micropolitan.

††† States whose reporting of any specific drug or drugs involved in an overdose changed by ≥10 percentage points from 2015 to 2016 were excluded because drug-specific overdose numbers and rates might have changed substantially from 2015 to 2016 as a result of changes in reporting.

§§§ Z-tests were used if the number of deaths was ≥100, and a p-value of <0.05 was considered to be statistically significant. Nonoverlapping confidence intervals based on the gamma method were used if the number of deaths was <100 in 2015 or 2016. Note that the method of comparing confidence intervals is a conservative method for statistical significance; caution should be observed when interpreting a nonsignificant difference when the lower and upper limits being compared overlap only slightly.

¶¶¶ Maps and figures providing significant changes in drug overdose and opioid-involved overdose death rates by state are available on CDC’s Drug Overdose website: https://www.cdc.gov/drugoverdose/data.

**** https://www.cdc.gov/nchs/data/databriefs/db294_table.pdf.

††††
https://www.cdc.gov/nchs/products/databriefs/db81.htm .

§§§§ 2017 National Drug Threat Assessment: https://www.dea.gov/docs/DIR-040-17_2017-NDTA.pdf; https://emergency.cdc.gov/han/han00384.asp; https://emergency.cdc.gov/han/han00395.asp.

¶¶¶¶ https://www.cdc.gov/nchs/data/series/sr_02/sr02_172.pdf.

***** https://www.cdc.gov/drugoverdose/foa/state-opioid-mm.html.

††††† https://www.cdc.gov/drugoverdose/prescribing/guideline.html.

§§§§§ https://www.cdc.gov/drugoverdose/states/state_prevention.html; https://www.cdc.gov/drugoverdose/foa/ddpi.html.
